# Potentiating the Heat Inactivation of *Escherichia coli* O157:H7 in Ground Beef Patties by Natural Antimicrobials

**DOI:** 10.3389/fmicb.2016.00015

**Published:** 2016-02-02

**Authors:** Meera Surendran Nair, Patrick Lau, Kaylin Belskie, Samantha Fancher, Chi-Hung Chen, Deepti Prasad Karumathil, Hsin-Bai Yin, Yanyan Liu, Fulin Ma, Indu Upadhyaya, Abhinav Upadhyay, Richard Mancini, Kumar Venkitanarayanan

**Affiliations:** Department of Animal Science, University of Connecticut, StorrsCT, USA

**Keywords:** *E. coli* O157:H7, ground beef, heat, resveratrol, rutin, chitosan

## Abstract

*Escherichia coli* O157: H7 (EHEC) is a major foodborne pathogen largely transmitted to humans through the consumption of undercooked ground beef. This study investigated the efficacy of two food-grade, plant-derived antimicrobials, namely rutin (RT), and resveratrol (RV) with or without chitosan (CH) in enhancing EHEC inactivation in undercooked hamburger patties. Further, the effect of aforementioned treatments on beef color and lipid oxidation was analyzed. Additionally, the deleterious effects of these antimicrobial treatments on EHEC was determined using scanning electron microscopy (SEM). Ground beef was inoculated with a five-strain mixture of EHEC (7.0 log CFU/g), followed by the addition of RT (0.05%, 0.1% w/w) or RV (0.1, 0.2% w/w) with or without CH (0.01% w/w). The meat was formed into patties (25 g) and stored at 4°C for 5 days. On days 1, 3, and 5, the patties were cooked (65°C, medium rare) and surviving EHEC was enumerated. The effect of these treatments on meat color and lipid oxidation during storage was also determined as per American Meat Science Association guidelines. The study was repeated three times with duplicate samples of each treatment. Both RT and RV enhanced the thermal destruction of EHEC, and reduced the pathogen load by at least 3 log CFU/g compared to control (*P* < 0.05). The combination of RT or RV with CH was found to be more effective, and reduced EHEC by 5 log CFU/g (*P* < 0.05). EHEC counts in uncooked patties did not decline during storage for 5 days (*P* > 0.05). Moreover, patties treated with RV plus CH were more color stable with higher a^∗^ values (*P* < 0.05). SEM results revealed that heat treatment with antimicrobials (CH + RV 0.2%) resulted in complete destruction of EHEC cells and extrusion of intracellular contents. Results suggest that the aforementioned antimicrobials could be used for enhancing the thermal inactivation of EHEC in undercooked patties; however, detailed sensory studies are warranted.

## Introduction

Enterohemorrhagic *Escherichia coli* O157: H7 (EHEC) is one of the major food-borne pathogens in the United States, causing an estimated 73,000 illnesses annually (Rangel et al., 2005). EHEC infection results in hemolytic uremic syndrome (HUS), characterized by hemolytic anemia, thrombocytopenia, and renal injury (Bell et al., 1997). Cattle are the principal reservoir of *E. coli* O157:H7, with fecal shedding being an important source of food and environmental contamination (Low et al., 2005). The majority of EHEC food-borne outbreaks in the US have been linked to the consumption of undercooked ground beef patties (Rhee et al., 2003; WHO, 2011), which is attributed to the thorough mixing of bacteria throughout the meat during grinding (Ferens and Hovde, 2011).

The United States Department of Agriculture (USDA) has established a zero tolerance policy for EHEC in ground beef, and recommended that beef patties be cooked to an internal temperature of 71.1°C (160°F) to ensure complete pathogen inactivation (USDA, 1998). Based on the degree of doneness, cooked patties can be classified as rare (60°C/140°F), medium-rare (65°C/149°F), medium (71.1°C/160°F) or well done (77°C/170.6°F) (Marksberry et al., 1993). A USDA survey on hamburger cooking practices in the US indicated that 20% of the participants cooked patties rare or medium rare (Ralston, 2002) which could result in EHEC survival in the meat. In order to ascertain that the required internal temperature (71.1°C) is attained during cooking of ground beef patties, the USDA advised consumers to use a meat thermometer. However, because of the lack of homogeneity in patty composition, and temperature monitoring difficulties, there is the possibility that the recommended internal temperature may not be uniformly attained (D’Sa et al., 2000). Further, the use of thermometers for cooking beef patties by consumers is limited (NCBA, 1999; McCurdy et al., 2005) due to the inconvenience of the procedure, consumer uncertainty, and a lack of consumer confidence in thermometer’s ability to ensure food safety (USDA, 1998; Research Triangle Institute [RTI], 2001; McCarty, 2008).

According to a survey conducted by the USDA-ERS, approximately 20% of the US population preferred eating rare or medium-rare patties at home, restaurants and cafeteria (Ralston, 2002). The same study revealed that at the internal cooking temperatures specified for rare and medium rare patties, EHEC numbers did not decline significantly compared to those cooked at 71.1°C. Moreover, most consumers determine the doneness of beef patties by observing the color and texture of cooked meat. However, color is not a good indicator of doneness because ground beef is prone to a non-typical color change associated with cooking called premature browning (PMB) (Claus, 2007), where the meat appears fully cooked in spite of not having achieved a safe internal temperature. Thus, PMB in ground beef can lead to inadequate cooking by consumers, who are misled by the cooked color (Warren et al., 1996a,b), potentially allowing EHEC survival. Killinger et al. (2000) reported that PMB incidence averaged about 47% in ground beef purchased from local retail stores. When compared to steaks and roasts, ground beef is more susceptible to PMB because of accelerated oxidation of the meat pigment, myoglobin that occurs as a result of grinding. The infectious dose of EHEC in humans is low (2–2,000 cells), emphasizing that undercooking of beef patties should be avoided (Buchanan, 1997; Strachan et al., 2005). Therefore, it is important to include an antimicrobial hurdle to ensure inactivation of EHEC in ground beef patties unintentionally cooked to inadequate temperatures.

Rutin (RT) is an antibacterial flavonoid obtained from buckwheat, rhubarb and berries (Kreft et al., 2006; Lupaşcu et al., 2008). Resveratrol (RV) is polyphenol found in grape, peanuts, dark chocolate and green tea with well-known antioxidant, anti-inflammatory and antimicrobial properties (Paulo et al., 2001; Sales and Resurreccion, 2014). Both these plant derived antimicrobials are generally recognized as safe (GRAS) and approved for use in foods (FDA, 2007, 2010). Chitosan (CH) is a GRAS-status polymer derived from the deacetylation of chitin, a natural polysaccharide present as the main component of exoskeletons of crustaceans (Kumar, 2000). CH possesses antimicrobial properties against a wide range of Gram-positive and Gram-negative bacteria (No et al., 2002; Sagoo et al., 2002). In addition, CH is used as an antimicrobial carrier coating or film on foods due to its emulsification and gelation properties (Knorr, 1984; Jiang and Li, 2001; No et al., 2007).

The primary objective of this study was to determine the efficacy of low concentrations of RT and RV for enhancing the inactivation of EHEC in undercooked ground beef patties (heated to 65°C, medium rare) in the presence or absence of CH. In addition, the effect of aforementioned treatments on meat color and lipid oxidation during refrigerated storage was determined.

## Materials and Methods

### Bacterial Strains

Five EHEC strains (E6, E8, E10, E16, and E22) all originated from cattle were used for the study. Strains E8, E10, and E16 were isolated from meat, strain E6 from milk and E22 was a calf feces isolate. All the five strains were induced for resistance to nalidixic acid (NA, 50 μg/ml, Sigma-Aldrich, St. Louis, MO, USA) for selective enumeration (Blackburn and Davies, 1994). Each strain was grown separately in 10 ml tryptic soy broth (TSB; Difco, Becton Dickinson, Sparks, MD, USA) supplemented with NA (Fischer Scientific, Pittsburgh, PA, USA) at 37°C for 24 h. After three consecutive transfers, all the three cultures were washed separately with phosphate buffered saline (PBS, pH 7.0) and suspended in PBS. Equal volumes of the five cultures were then combined and pelleted by centrifugation at 3600 × *g* for 15 min at 4°C. The pellet was suspended in 10 ml of PBS and the bacterial suspension was used as the inoculum. The EHEC count in the inoculum was determined by serially dilution (1:10 in PBS) and plating on tryptic soy agar (TSA; Difco) plates containing NA (50 μg/ml) and incubating the plates at 37°C for 24 h.

### Preparation of Ground Beef Patties

Fresh fine ground beef (80% lean and 20% fat) was purchased from a local meat processor. The ground meat was separated into portions of 25 g each, and randomly assigned to different treatments. Each meat portion was inoculated with the five-strain mixture of EHEC (∼7.0 log CFU/g), followed by the addition of RT (0.05%, 0.1%w/w, Sigma–Aldrich, MW: 610.52, soluble in DMSO and Ethanol), or RV (0.1, 0.2%w/w, Candlewood Stars Inc., Danbury CT, USA, MW: 228.24, soluble in DMSO and Ethanol), with or without CH (0.01%w/w, Sigma–Aldrich, MW: ∼1.5 KDa). CH (1%, w/v) stock solution was prepared in 1% acetic acid solution to dissolve RV. The ground beef was mixed thoroughly and formed into patties (25 g). The patties were then placed on foam trays and wrapped with oxygen-permeable fresh meat film (Koch Supplies, Kansas City, MO, USA) and stored at 4°C for 5 days.

### Cooking

On days 0, 1, 3, and 5 of storage, the inoculated patties were cooked separately in a double-sided George Foreman Lean Mean Grilling Machine (Salton Inc., Columbia, MO, USA) until an internal temperature of 65°C (medium rare) was reached. A standard meat thermometer (Acutuff Model 34 Atkins 2 mm probe meat thermometer, Koch Supplies) was used to monitor the internal temperature continuously. In order to ensure uniform cooking on either sides, the patties were turned thrice during cooking (at 30°C, at 40°C, and at 50°C). The entire cooking procedure was done based on a published protocol (Suman et al., 2005; Amalaradjou et al., 2010). In addition, a set of uncooked, refrigerated patties containing each treatment was included to study the effect of aforementioned antimicrobials on EHEC without heat.

### Bacterial Enumeration

Each cooked patty was immediately transferred to a sterile Whirlpak bag (Nasco, Fort Atkinson, WI, USA) containing 30 ml of sterile ice-cold neutralizing broth (Dey–Engley neutralizing broth, Sigma–Aldrich), and homogenized in a stomacher for 2 min at high speed. The surviving EHEC population was enumerated by spread plating 100 μl of serially diluted homogenate on duplicate TSA plates supplemented with NA (50 μg/ml). In addition, 1 ml of the meat homogenate was added to 9 ml of TSB containing NA and incubated at 37°C for 24 h. Following incubation, the culture was streak plated onto TSA plates supplemented with NA (50 μg/ml) and Sorbitol MacConkey agar supplemented with 4-Methylumbelliferyl-β-D-glucuronide (Oxoid Ltd, Lenexa, KS, USA).

### Meat Color

Instrumental color measurements were done to determine the effect of the antimicrobial treatments on meat color (Hunt et al., 1991, 2012; Amalaradjou et al., 2010; Suman et al., 2010). Using HunterLab MiniScan XE Plus colorimeter (HunterLab Associates, Reston, VA, USA) with illuminant A, 2.54-cm diameter aperture, and 10° standard observer, a^∗^ values and reflectance spectra from 400 to 700 nm at 10 nm increments were measured on patties on days 0, 1, 3, and 5 days of storage.

### Lipid Oxidation

The effect of RT and RV with or without CH on lipid oxidation in patties was determined using a procedure previously described (Sinnhuber and Yu, 1958; Hunt et al., 1991, 2012). Briefly, 0.5 g of minced portion of each ground beef patty was added with 2.5 ml of thiobarbituric acid (TBA; Sigma–Aldrich) stock solution (thiobarbituric acid, 15% trichloroacetic acid, and 0.25 N HCl) to get a dilution factor of 6. After proper mixing, the samples were heated in a boiling water bath for 10 min. As soon as the samples from the control patty (without treatments) turned pink, the samples were immediately washed in tap water to cool down. The absorbance spectra of the clear supernatant obtained by centrifuging the cooled samples at 5,000 × *g* for 10 min was measured at 532 nm against a blank containing reagents alone (without meat). The thiobarbituric acid reactive substances (TBARS) value in ppm for each sample was determined separately using the equation: TBARS value (ppm) = sample A532 × 2.77.

### Scanning Electron Microscopy

Scanning electron microscopy (SEM) was done to visualize the deleterious effects caused by thermal destruction and RV with or without CH on EHEC cell morphology. Since RV was found to be more effective than RT in inactivating the pathogen, SEM was done only for samples treated with RV. All the five strains of EHEC were grown separately in 10 ml TSB (Difco, Becton Dickinson) at 37°C overnight. Approximately 7 log CFU/ml of the five strain EHEC mixture in TSB was treated with RV (0.2%) with or without CH (0.01%) and heated at 65°C for 120 s. Appropriate heated and unheated controls were also included. After the treatment, the bacterial cells were washed immediately in PBS, pelleted out by centrifuging at 3,500 *g* for 10 min, and suspended in 1 ml of PBS. The samples were then deposited onto POLY-L-LYSINE coated silicon wafer chips (Prod No. 16008, Ted Pella Inc.) and bathed in fixative solution (1.5% paraformaldehyde, 2% glutaraldehyde, in 0.1 M sodium cacodylate, pH 7). After incubation in fixative at 4°C for 90 min, the cells were washed with 0.1 M Na cacodylate buffer (pH 7) and post-fixed in 1% osmium tetroxide at 4°C overnight. The cells were rinsed twice for 5 min in distilled water then dehydrated in serial concentrations of ethanol (30, 50, 70, 95, 100, 100% ETOH, 5 min each), and critical point dried (931GL, Tousimis). The dried cell samples were then mounted on SEM stub using silver paint and sputter coated with gold/Palladium (E5100, Polaron)and examined using a scanning electron microscope (Nova NanoSEM 450, FEI) (Woo et al., 2000; Moussa et al., 2007).

### Statistical analysis

The inactivation study was a split plot with completely randomized design. Each batch of ground beef was divided into 240 patties of 25 g each. The patties were randomly assigned to 1 of 40 of the treatments^∗^storage time combination effects. Each patty served as an experimental unit and the entire study repeated three times with duplicate samples. The data were analyzed using GLIMMIX procedure of SAS 9.4 and significance was tested at *P* < 0.05. Similar analysis was done for the TBARS estimation. The a^∗^ values were analyzed as repeated measures using proc mixed procedure, and the experimental design was a randomized complete block design. The significance was tested at *P* < 0.05.

## Results

**FIGURE 1:**
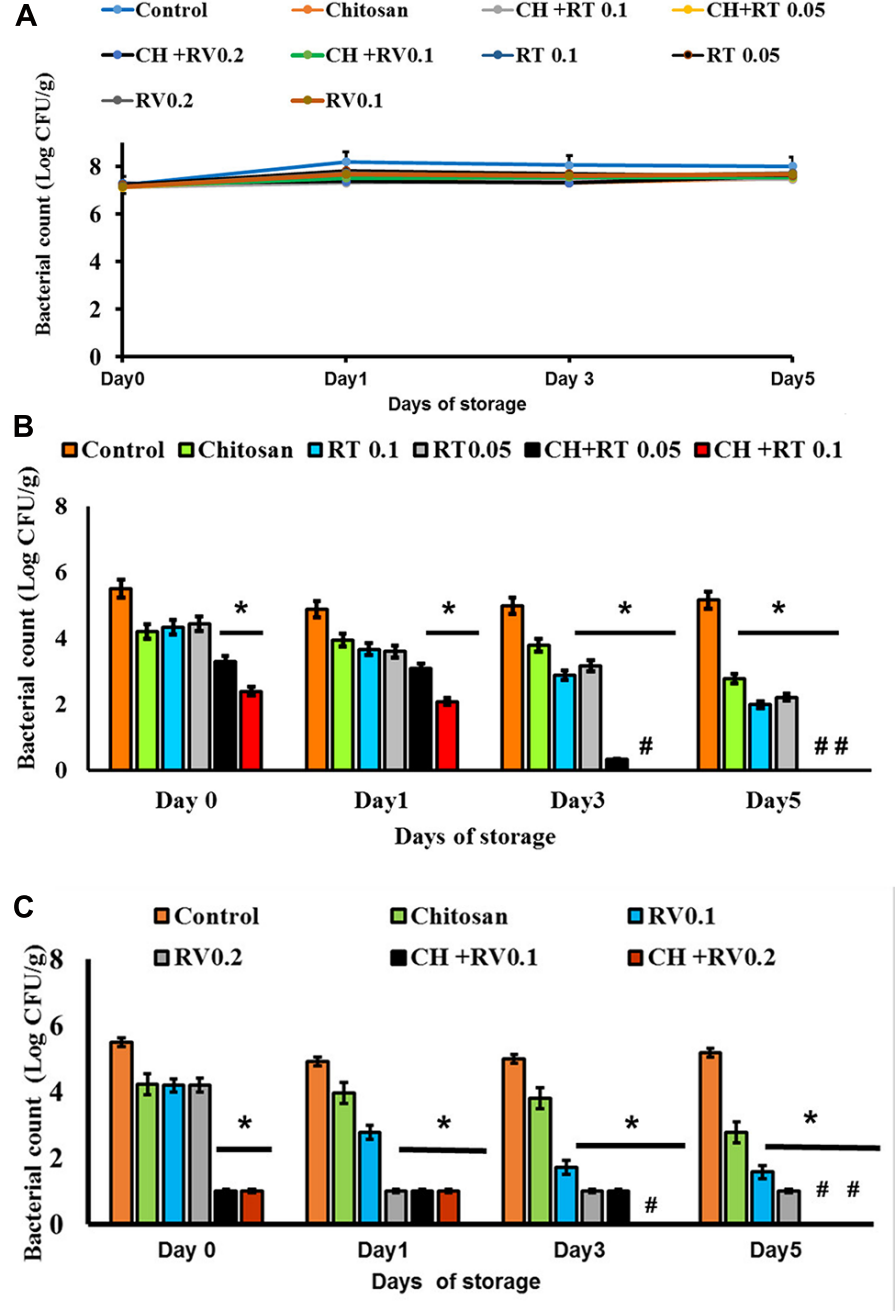
**(A)** Effect of Rutin (RT) and Resveratrol (RV) with or without Chitosan (CH) on EHEC in uncooked ground beef patties stored at 4°C for 5 days. **(B)** Effect of RT with or without CH on EHEC in ground beef patties stored at 4°C for 5 days and cooked to an internal temperature of 65°C. **(C)** Effect of RV with or without CH on EHEC in ground beef patties stored at 4°C for 5 days and cooked to an internal temperature of 65°C. # – Negative by Enrichment. ^∗^ Treatments were significantly different from the control at *P* < 0.05.

The effect of RT and RV with or without CH in potentiating the thermal inactivation of EHEC in undercooked ground beef patties is depicted in **Figure 1**. There was no decline in EHEC counts irrespective of the treatments throughout the refrigerated storage in uncooked patties, and ∼6.5 log CFU/g of the pathogen was recovered from patties at the end of storage period (*P* > 0.05) (**Figure 1A**). However, when the patties were cooked to 65°C, the EHEC population was decreased by ∼1.5 to 2.0 log CFU/g in untreated control patties. On the other hand, both tested concentrations of RT in combination with CH decreased EHEC counts by 2–3.0 log CFU/g on days 0 and 1, with >5.0 log CFU/g reduction in bacterial counts on days 3 and 5 of storage (enrichment negative; *P* < 0.05) (**Figure 1B**). Similarly, both concentrations of RV in combination with CH reduced EHEC counts by >3 log CFU/g, with no pathogen detected in the patties containing 0.2% RV plus CH on days 3 and 5 of storage (*P* < 0.05) (**Figure 1C**).

**FIGURE 2:**
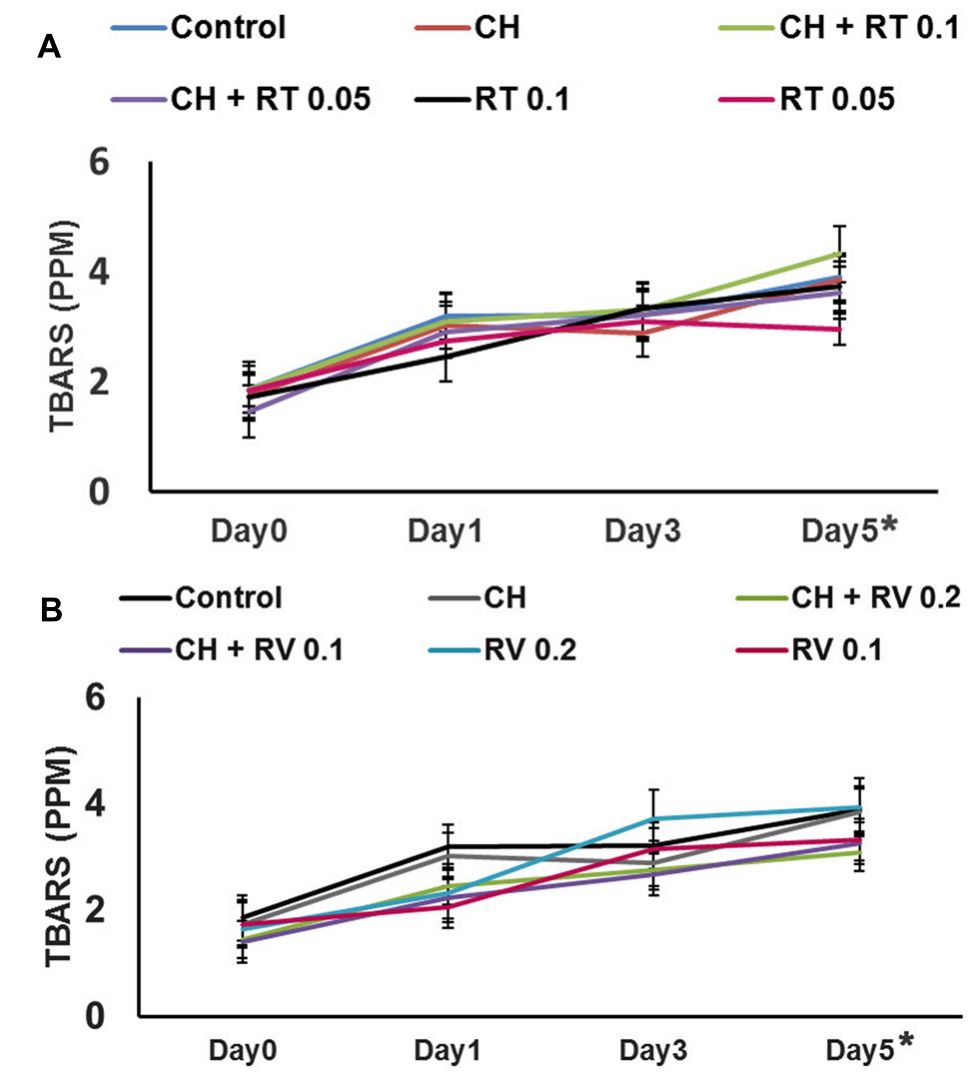
**(A)** Effect of Rutin (RT) with or without Chitosan (CH) on lipid oxidation in ground beef patties stored at 4°C for 5 days. **(B)** Effect of Resveratrol (RV) with or without CH on lipid oxidation in ground beef patties stored at 4°C for 5 days. Higher values of TBARS denote more lipid oxidation. There was no significant difference in lipid oxidation observed between the treatments and control on any day of storage (*P* > 0.05). However, the TBARS values increased significantly from day 0 to day 5, irrespective of the treatments (*P* < 0.05) (indicated by ^∗^).

The instrumental color measurements for redness of meat (a^∗^ values) are shown in **Table 1**. As expected, the red color of control and treated patties gradually decreased over time during storage. The patties treated with RV plus CH exhibited greater redness (*P* < 0.05) throughout the storage compared to the control. However, there was no difference between the a^∗^ values of CH only containing patties and control patties except on day 3 (*P* > 0.05). In addition, the patties treated with RT (0.1 and 0.05%) plus CH displayed greater a^∗^ values on day 3 of storage compared to control patties (*P* < 0.05). The formation of TBARS in beef patties was determined on days 0, 1, 3, and 5 of storage. Although TBARS increased progressively in patties during storage (*P* < 0.05), no significant difference in lipid oxidation was observed between the treatments on any day of storage (*P* > 0.05) (**Figure 2**).

**Table 1:** Effect of RT and RV with or without CH on meat color in ground beef patties stored at 4°C for 5 days.

Treatment	a^∗^ value ±SE			
	Day 0	Day 1	Day 3	Day 5
Control	29.81 ± 0.84^ab^	25.23 ± 0.71^bc^	17.32 ± 0.62^f^	12.08 ± 0.95^bc^
CH	30.26 ± 0.94^ab^	26.67 ± 0.59^ab^	21.72 ± 0.54^abc^	12.35 ± 0.92^bc^
CH + RT 0.1	29.79 ± 0.94^ab^	26.13 ± 0.41^b^	21.54 ± 0.37^abcd^	12.04 ± 0.84^bc^
CH + RT 0.05	29.87 ± 1.27^ab^	26.06 ± 0.44^b^	20.95 ± 0.67^bcde^	13.57 ± 0.95^bc^
CH + RV 0.2	31.45 ± 0.48^a^	30.02 ± 0.64^a^	23.89 ± 0.27^a^	16.44 ± 0.9^a^
CH + RV 0.1	30.78 ± 0.75^ab^	29.84 ± 0.82^a^	22.69 ± 0.47^ab^	17.85 ± 0.96^a^
RT 0.1	29.84 ± 0.82^ab^	28.24 + 0.26^bc^	20.01 ± 0.96^cde^	15.23 ± 0.93^ab^
RT 0.05	28.66 ± 0.71^b^	27.85 ± 0.76^c^	18.62 ± 0.88^ef^	14.08 ± 0.98^abc^
RV 0.2	30.02 ± 0.64^ab^	27.02 ± 0.64^bc^	18.94 ± 0.91^def^	14.55 ± 0.93^abc^
RV 0.1	29.67 ± 0.69^ab^	29.67 ± 0.69^bc^	18.68 ± 0.96^ef^	12.90 ± 0.92^bc^

*Increased a^∗^ value indicated increased redness of meat and means with same letters are not significant down each column.*

**FIGURE 3:**
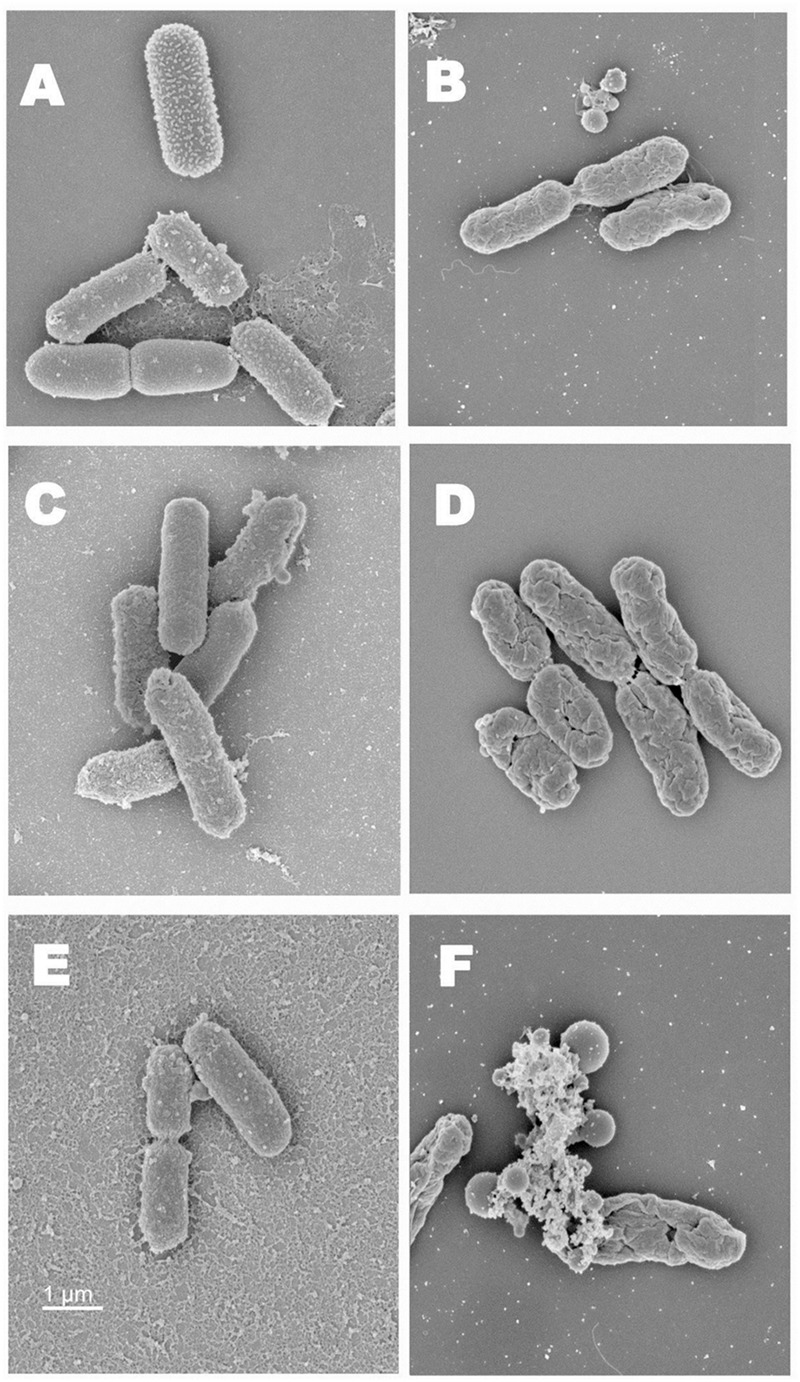
**(A)** Scanning electron microscopy images of normal EHEC cells (Five-strain cocktail). **(B)** SEM images of EHEC cells exposed to heat. **(C)** SEM images of EHEC cells treated with RV (0.2%). **(D)** SEM images of RV treated EHEC cells exposed to heat. **(E)** SEM images of EHEC cells treated with RV in CH. **(F)** SEM images of RV + CH treated bacterial cells exposed to heat.

Scanning electron microscopy imaging of EHEC cells exposed to heat and the antimicrobials revealed structural changes on bacterial cell surface. As shown in **Figures 3A,B**, compared to unheated control, EHEC cells that were subjected to heating alone (65°C) were found to be dehydrated with evident surface changes. The heat exposed and RV treated bacterial cells were dehydrated and had pores on their cell surface (**Figures 3C,D**). Furthermore, the combination of heat and antimicrobials (CH + RV 0.2%) caused a complete destruction of bacterial cells with pore formation and extrusion of intracellular exudates (**Figures 3E,F**). In addition, the bacterial cells appeared to be more spherical than rod shaped, occasionally with some bleb like structures.

## Discussion

This study investigated the effect of two food-grade plant-derived antimicrobials, namely RV and RT in enhancing the thermal inactivation of EHEC in undercooked ground beef patties in the presence and absence of CH. CH, a known emulsifier, was primarily used for the uniform distribution of the antimicrobials in meat. It was observed that RT or RV alone or in combination with CH did not exert any antimicrobial effect on EHEC in uncooked refrigerated ground beef (**Figure 1A**). However, when the patties were heated to an internal temperature of 65°C, both plant compounds in combination with CH brought about significant reductions in EHEC counts throughout the storage period (*P* < 0.05) when compared to control and patties containing RT, RV, or CH alone. These results indicate that the combination of RT or RV with CH and heat had a greater lethality on EHEC in beef patties than each of the antimicrobials or heat alone. The increased heat destruction of EHEC in patties containing the antimicrobials could be attributed to their pronounced deleterious effects on bacterial cell membrane leading to the loss of intracellular contents and complete destruction of the cells, as evident from the SEM results (**Figures 3A–F**). In addition, heat-induced damage of bacterial plasma membrane may have potentially allowed rapid accumulation of the antimicrobials within the cells, thereby resulting in an enhanced bactericidal effect. For example, Shibasaki and Kato (1978) observed that heating causes bacterial plasma membrane to become more fluid in nature, thereby increasing the antimicrobial activity of lipid-soluble small molecules. Antimicrobial flavonoids, including rutin have been reported to act on lipid bilayers of bacteria and believed to modify the physicochemical properties of the cell membranes (Tsuchiya, 2015). They are also known to interact with functional protein systems of the cell (Koval’skii et al., 2014; Tsuchiya, 2015). Similarly, several studies showed the deleterious effect of RV on bacterial membrane, which includes oxidative damage to the cell membrane, loss in membrane integrity and morphology (Bajpai et al., 2014; Subramanian et al., 2014; Taylor et al., 2014).

It was also observed in the study that CH by itself did not result in a significant reduction in EHEC counts (*P* > 0.05), except in patties that were cooked after 5 days of refrigerated storage (**Figures 1B,C**). This was expected since the concentration of CH used in the study (0.01% w/w) was much below the antimicrobial concentration (0.5–1%) reported in the literature (Darmadji and Izumimoto, 1994a,b). However, the combination of RT or RV with CH increased the thermal destruction of EHEC, which could be attributed to the improved solubility and distribution of the antimicrobials in the presence of the CH, which is a potent emulsifier (Schulz et al., 1998; Klinkesorn, 2013) and solubilizer (Zerrouk et al., 2004; Shete et al., 2015). In addition, synergistic antimicrobial effects between CH and a variety of antimicrobials, including essential oils (Wang et al., 2011) have been observed, and this could have contributed to the enhanced bacterial killing in cooked patties containing RT or RV with CH. Previously, two plant-derived compounds, namely carvacrol and trans-cinnamaldehyde were found to enhance the thermal destruction of EHEC in ground beef (Juneja and Friedman, 2008), where addition of both compounds in beef sensitized the pathogen to the lethal effect of heat, as evident from the significantly lower thermal destruction times of the pathogen in the treated samples. Similarly Amalaradjou et al. (2010) reported that addition of trans-cinnamaldehyde (0.15, and 0.3%) significantly reduced EHEC counts in ground beef cooked to an internal temperature of 60 or 65°C compared to untreated patties cooked to the same temperatures. Yet another study found that incorporation of apple skin and tea leaf powders at 3% level in ground beef decreased the heat resistance of EHEC in Sous-wide cooked ground beef (Juneja et al., 2009).

Since the attractive red color of meat is the primary yardstick by which consumer purchasing decisions of beef and beef products are made (Mancini and Hunt, 2005), we determined the effect of RT, RV and CH on the color of uncooked beef patties during refrigerated storage. The results from meat color analysis revealed although none of the treatments adversely affected meat color compared to control patties; the beef patties treated with RV and CH displayed a greater red color (a^∗^ values) throughout the storage period (*P* < 0.05). Further, patties containing RT and CH demonstrated a greater redness on days 3 and 5 of storage compared to control patties (*P* < 0.05). Previous studies have revealed that CH at 1% increased the redness and color stability of frozen beef patties and pork sausages (Lin and Chao, 2001; Georgantelis et al., 2007a). However, in this study a much lower concentration of 0.01% (w/w) was used and the color of patties containing only CH was not significantly greater than that of control samples. Moreover, the aforementioned researchers observed an elevated pH in CH added meat in their studies, and the increased meat pH may have minimized myoglobin oxidation and surface discoloration, thereby maintaining the meat color stability (Suman et al., 2010). However, we did not observe any significant change in the pH of meat irrespective of the treatments throughout the study. Therefore the mechanism behind the increased redness in patties with RV or RT in combination with CH needs to be investigated.

A primary cause for quality deterioration of meat during storage is lipid oxidation, which adversely affects color, flavor, texture and nutritional value of meat. Previous studies have shown that plant antimicrobials such as trans-cinnamaldehyde and rosemary decreased lipid oxidation in beef patties and pork sausages (Georgantelis et al., 2007b; Amalaradjou et al., 2010). In addition, CH at doses ranging from 0.2 to 1% has been documented for its ability to minimize lipid oxidation in meat. However, we did not observe any protective effect of RT, RV or CH in reducing lipid oxidation in refrigerated beef patties, and the amount of TBARS was not different among patties throughout the storage period (*P* > 0.05).

## Conclusion

Results of this study indicate that RV and RT in the presence of CH significantly increased the heat inactivation of EHEC in undercooked ground beef patties without adversely affecting meat color and lipid oxidation. Therefore, these natural antimicrobials could be added to ground beef for improving the microbiological safety of undercooked beef. However, organoleptic studies are warranted for ascertaining the consumer acceptability of patties supplemented with the aforementioned additives.

## Author Contributions

KV is the corresponding author and primary contact during manuscript submission, review and publication process. The work was done under his supervision as the principal investigator. He significantly contributed to the design, drafting, revisions and interpretation of data. The manuscript is being submitted with his final approval for publication. MN is the submitting author and accountable for all parts of the work done and for questions related to accuracy and integrity of the entire work. She is the major player in the conception, design, conduct, revision, analysis and interpretation. The manuscript is being submitted with her final approval for publication. PL, KB, SF, C-HC, DP, H-BY, YL, FM, IU, AU and RM contributed to the conduct of different sections of the entire work. They agreed to be accountable to the different parts of the work and approved the final version for submission.

## Conflict of Interest Statement

The authors declare that the research was conducted in the absence of any commercial or financial relationships that could be construed as a potential conflict of interest.
